# Science fiction and ELSI: three thoughts

**DOI:** 10.3389/fgene.2023.1270590

**Published:** 2023-12-11

**Authors:** Henry T. Greely

**Affiliations:** ^1^ Stanford Law School, Stanford University, Stanford, CA, United States; ^2^ Center for Law and the Biosciences, Stanford, CA, United States

**Keywords:** science fiction, fantasy, ethical, legal, and social issues, ethical, legal, social

## Abstract

Science fiction can be useful to those who analyze ethical, legal, and social issues (ELSI) in genetics and the biosciences more broadly. It can provide examples of possible technological changes, which are occasionally valuable as predictions of the future but more often helpful as indicators of the likely social consequences of such technologies. This “what-if” approach to science fiction can also provide a good pathway to exploring such issues. Science fiction can also allow a more distant, less realistic, and non-culture-specific context for exploring deep questions about humanity, ethics, and other major issues. At the same time, science fiction also has some negative effects on such analysis or its reception as a result of the need for fiction to hold its audience by providing drama through conflict. This necessity for successful fiction often leads to technological or cultural changes being portrayed as catastrophic and dystopian, much more often than beneficial or utopian. This imbalance can predispose public opinion against innovations unfairly, in part by providing “examples” from fiction of similar innovations, leading to bad outcomes. ELSI researchers should keep this fiction-induced bias in mind in their work.

## 1 Introduction

For me, it started over 60 years ago, in Oz. I was probably seven or eight. My father’s mother had kept some old Oz books from her children’s youth, and they had made their way to our home. I remember that the book was beautiful—hard-bound with gorgeous, full-page, and color illustrations, probably editions published in the 1930s. I do not remember which of the 40 Oz books I read first, but I was hooked.

Oz led me to John Carter, Jeddak of Jeddaks of Barsoom. I jumped from Burroughs to Robert A. Heinlein and then kept moving onward and outward. I was a gluttonous and wide-ranging reader but, at least through college, I mainly read science fiction and, eventually, fantasy. (I will hereafter refer to these two related but different genres, for the ease of reference, as “science fiction.”) I have continued to read and watch science fiction and fantasy for the last 50 years, although it now accounts for a smaller fraction of my total reading.

That passion for science fiction is a major reason I ended up doing the work I do, trying to understand the ethical, legal, social, policy^1^, and political implications of advances in the biosciences (ELSI). I am not a literary scholar of any kind,^1^ let alone one working in the burgeoning field of science fiction and fantasy studies,^2^ but, over the years, my work has given me some insights into how this literature affects my field. I cannot guarantee that the ideas in this article are novel, but I at least had not seen them before.

This article discusses two specific kinds of science fiction that can help ELSI discussions: science fiction that projects future developments and their social consequences and science fiction that explores important ideas. However, its third point is that science fiction can also cause significant problems owing to fiction’s inevitable bias toward conflict, a bias with social consequences.

## 2 “What if?”

Science fiction has a very mixed record on actually predicting future technologies. Little science fiction predicted personal computers, social media, or smart phones, and much that it predicted has not transpired. However, science fiction can provide a framework for exploring the ramifications of changes. I think of these as “what-if” stories. If this technological change is thrown into society, what ripples—or tsunamis—does it cause? These changes flesh out the interplay between the new technology and how society will react to it. Neither the new technologies nor the exact reactions to them are likely to prove very accurate, but they can give some ideas for the types of reactions one might look for and look out for.

In his early career, Heinlein was a master of “what-if” stories. Three examples of his contributions are as follows: in his short story, *Let There Be Light* (Heinlein, 1940), his protagonists invent a highly efficient lighting system, only to realize that it can also run in reverse as a solar electricity generator. I do not know if this is the first time solar panels have appeared in science fiction, but the social reaction is interesting. Power companies do not like it and attempt to eliminate it. Therefore, our heroes publish the details and make it publicly available for a small royalty (arguably prefiguring Stanford University’s licensing strategy 40 years later for its recombinant DNA patents).

In his short story, *Jerry Was a Man* ^3^ (Heinlein, 1947), the plot revolves around genetic engineering, albeit with murky science (fair enough as Crick and Watson’s paper on the structure of DNA was still 6 years in the future). The story features a pet dwarf elephant, a winged (but flightless) horse, and intellectually enhanced chimpanzees (“humanzees?”) that were widely used as unskilled laborers. The plot revolves around whether or not one of those chimps, called Jerry, was entitled to human rights.

*Methuselah’s Children* (Heinlein, 1958) is another example for Heinlein’s early books of science fiction. The 22nd-century “Howard Families” face a problem. They have extremely long lives, often exceeding 150 years, but only as a result of genetic conditions (and matchmaking). They have lived in fear of the envy of the other 99% of the world’s population and so have kept themselves hidden, “dying,” and re-appearing under a new identity elsewhere when their failure to age becomes too obvious. The masquerade eventually fails, and the world persecutes them for their (non-existent) technological secret. How would our society react if a small minority of its people lived longer or had greater health than the rest? I have based final exam questions on modern and more realistic variants of this scenario.

Examples are near infinite because much science fiction works this way. ELSI scholars may find that science fiction has already done some speculation about their issues, explorations that may lead in different directions. Thus, it may be useful to do a literature search not only in the scholarly literature but also in science fiction. At least one author has suggested something similar, with respect to ELSI in nanotechnology (Lopez). However, more fundamentally, science fiction here provides both a template and a kind of “permission” for ELSI researchers to do the same with innovations that are already beginning or viewed as clearly plausible.

My own work has often been based on the same idea: “what if” this technology were to be widely used? How would that affect our societies and ourselves? My book, *The End of Sex and the Future of Human Reproduction* (Greely, 2016), was based on such a question, although not initially asked by me. In October 2010, I was at a stem cell conference in Münster, Germany. Laurie Zoloth presented a speech focusing on “induced pluripotent stem cells” (iPSCs), a recently invented technology that would make undifferentiated cells, similar to human embryonic stem cells, from differentiated cells, such as skin cells. In her speech, Zoloth had one sentence asking “What if these cells could be turned into eggs and sperm?”

“Hmm,” I thought, what would follow from that? Six years later, I had some answers and a book on “*in vitro* gametogenesis”—making eggs and sperm from skin cells—that led me far from where I had expected to go.

I have written many articles based on that same inquiry. However, the “what-if” approach to ELSI does have some limitations. It necessarily attempts to predict the future, which is easy yet very hard to do accurately. I want my scholarly work to be non-fictional and projecting too far ahead seems to be of little value to me. I will not write about actual human immortality or “uploading” human personalities, for example, as I think they are far away, perhaps infinitely so.

## 3 Exploring ideas

I remember a conversation many years ago with another ELSI scholar who loves science fiction. We were comparing favorite authors, and she said something like “you like world building and I like playing with ideas.” As a result, hers is another way science fiction that can be useful in ELSI work; it can explore ideas, unbounded by the need to be relatively plausible.

Ursula Kroeber Le Guin was great at this approach. Three of her works in particular use fictional settings to play with questions of culture and ethics.

In *The Dispossessed*, Le Guin (1974) tells the story of a scientist born and raised in an anarchist society, in exile on a planet’s habitable moon, who migrates to the planet where very different cultures exist. It is a hard-eyed exploration of the philosophy of anarchy and how it might work, or not, if seriously tried.

In *The Left Hand of Darkness* (Le Guin, 1969), she looks at a different kind of culture in a world where the humans have no fixed sex but can change physiologically between male and female monthly. What would a world without fixed gender be like, not just in its organization but in its society and interpersonal relations?

And, to me, *The Ones Who Walk Away from Omelas* (Le Guin, 1973) was her best story. The story is set in a coastal town that appears to be not too different from modern America, except that Omelas is a perfect town, a true utopia; except for the fact that its perfection has a price.

Their happiness, the beauty of their city, the tenderness of their friendships, the health of their children, the wisdom of their scholars, the skill of their makers, even the abundance of their harvest, and the kindly weathers of their skies, depend wholly on [a] child’s abominable misery.

The story asks, how do the people of Omelas react?

This idea is not new; the child is a kind of scapegoat, found in the book of Leviticus, when a goat chosen on Yom Kippur was to be sent into the wilderness and killed, to remove some of the sins of the people (The Bible). Le Guin said her story was inspired by an essay by philosopher William James, although she admitted that it may also have been affected by half-forgotten memories of Dostoyevsky’s *The Brothers Karamazov* (Wikipedia). Shirley Jackson’s famous 1948 short story, *The Lottery* (Jackson, 1948), is another sibling. However, I would argue that the dreamy, real but unreal setting that science fiction allowed Le Guin, along with her own exquisite writing, gave her version great power. Therefore, I think reading it can help ELSI scholars appreciate anew some questions of justice.

In another slightly different way, Frank Herbert’s *Dune* inspired new thinking (Herbert, 1965). The universe of *Dune* encompassed many ideas, from religion to psychedelics, but ecology, and, more broadly, environmentalism, were central to the story of the desert world, Arrakis, and its inhabitants, the Fremen. Their water conservation measures and their careful and slow efforts to transform their planet presented a powerful view of another type of environmental thinking based both on environmental science and Herbert’s discussions with Native Americans.

*Dune* appeared at the very beginning of the modern environmental movement, which was arguably started by the September 1962 publication of Rachel Carson’s *Silent Spring* (Carson, 1962). Although environmentalism (and its conservationist predecessor) had roots well before the 1960s, that decade saw its growth, flourishing, and, in the United States, its first major legislative successes. Did *Dune* cause the environmental movement? Of course not. However, its readers came away with ideas that contributed to growth of that movement.

ELSI scholars can similarly approach science fiction for ideas and their consequences that may affect the future uses of new biotechnologies.

## 4 But…the demands of the narrative

Science fiction can help the analysis of ELSI questions, but it can also harm it, in the thoughts of the analysts, but perhaps more importantly, in the public reception of that analysis.

Science fiction authors generally need to write stories for which they will be paid, which means stories that editors think people will pay to read. Although readers may find different things particularly pleasing in fiction, they all (or almost all) want a good story. They want a narrative with a beginning, a middle, and an end, in which interesting things happen to engaging (good or bad) characters. And good stories almost always involve conflict. It may be the conflict with Sauron for the future of Middle Earth; it might be the conflict with the emperor and the Harkonnens for the future of Arrakis and the empire; the conflict between the Rebel Alliance and the empire for the freedom of the Galaxy; or the Howard Families and humans jealous of their longevity. However, there (almost) always has to be conflict.

In science fiction, conflicts often revolve around the things, technological or fantastic, that make the author’s world different. Therefore, the easy and tempting path will be to base the story on conflicts caused by the new technology or the fantastic differences.

One need only look at three of the most well-known early books of science fiction: Mary Wollstonecraft Shelley’s *Frankenstein* (Shelly, 2018), Aldous Huxley’s *Brave New World* (Huxley, 1932), and, I would argue, George Orwell’s *1984* (Orwell, 1949). Each book involves technological changes that have terrible consequences. Although I referred only slightly to movies or television in this article, memorable visual science fiction often follows a similar path. When I talk about genetic selection or editing, someone in the audience will inevitably bring up, as an argument against the technology, the film *Gattaca* (Columbia Pictures, 1997). When I talk about de-extinction, I get told “remember what happened in *Jurassic Park*!” (Crichton, 1990; Universal Pictures, 1993). To which I respond, “remember, that was fiction.”

In the real world, dystopias can happen as the 20th century more than demonstrated. Yet, most of the time, we do not end up in dystopias. We never receive utopias, but we usually muddle through. Muddling through lacks drama. It is not exciting, and it does not sell books or tickets. Therefore, fiction presents future technologies as leading to dystopian horrors; no one writes successful science fiction about utopias.

To have the conflict revolve around apparently attractive changes that cause catastrophe fits well with some deep strands in Western cultures. It often ties to the idea of “forbidden knowledge,” things that “man is not meant to know.” In the Judeo-Christian component of Western culture, this can be traced back to the literal beginning, in the aptly named Genesis. Adam and Eve succumb to the temptation to taste the fruit of the tree of knowledge of good and evil and they, along with all their descendants, are punished for it. The Greek root of Western civilization has a similar myth. Prometheus disobeyed Zeus and brought fire to humans. To punish humanity, Zeus sent Pandora to be the wife of Prometheus’s brother. With her went a jar (mutated over the years through various translations into a box) that she was warned not to open. When she did, great evils were unleashed upon the world.

In both stories, humanity suffers, forever, from their disobedience to divine commands. However, it is not just humanity as a whole that is punished. So are those who did the deeds, from Adam and Eve, expelled from paradise, to Prometheus, bound to a mountain top where his liver was eaten out of his body by an eagle every day but would regenerate every night.

These types of myths are not merely ancient history. Philip Ball has written about modern myths, stories that modern fiction regularly returns to. His book, *The Modern Myths: Adventures in the Machinery of the Popular Imagination* (Ball, 2021), examines seven such myths. Although they range from Robinson Crusoe to Batman, two come directly from science fiction: *Frankenstein*, with its dreams (and fears) of reanimation, and H.G. Wells’s *The War of the Worlds* (Wells, 1898), with its nightmare of invasion by “the others.”

Pure dystopias are not common in contemporary literature and movies. Instead, our protagonist usually manages to escape from the dystopia or its worst effects, sometimes with stunning victories (most of the *Star Wars* movies), or other times by making a narrow escape, as in *Gattaca*, though often with the sequel-generating possibility that the evil will return (*Jurassic Park*).

Evidently, not all science fiction deals with new technologies or spread fear of divine retribution for human presumption. Much of it only tells engaging stories set in very different, and interesting, environments. In some unusual cases, the world approaches a utopia: the post-scarcity and peaceful United Federation of Planets in the original *Star Trek* series, as imagined by its creator, Gene Roddenberry, seemed almost too good to be true. That aspect engendered conflict between Roddenberry and writers, directors, and network, who (rightly) concluded that the series needed conflict in order to hold viewers’ interest, leading to the Klingons and other alien threats.

However, much science fiction, particularly well-known science fiction, taps into these myths. Almost all new bioscience (and non-bioscience—artificial intelligence certainly has been often explored in science fiction) has been explored in frightening ways in science fiction.

As a result, people disturbed or concerned by bioscience discoveries or inventions will often not have to look hard for well-known fictional “examples” of the evils that those discoveries might create. Of course, science fiction is not the only source of such negative examples. Every discovery or action in human genetics is greeted with warnings of a new “eugenics,” a case where, unhappily, history proved as bad as fiction. However, science fiction certainly contributes to uneasiness about new developments, providing popular arguments and feeding popular movements.

This is not an entirely bad thing. We *should* worry about the consequences of new technologies that are especially true in biosciences because of both the complexities of biology and the difficulties of controlling living, often moving, and reproducing organisms.

The problem is that fiction is unbalanced. Because it needs conflict, it offers many problems stemming from new technologies, but few examples of their good uses. As far as I know, no one has written fiction using Victor Frankenstein’s methods to provide new arms, legs, or organs for sick and maimed people. To the best of my knowledge, no one has shown happy and excited crowds safely roaming Jurassic (more plausibly, Pleistocene) Park, emerging with more knowledge and, for some, with a new focus for their studies and careers.

ELSI scholars need to keep the lessons to draw from the fiction in context, both for themselves and for their readers. Fiction does not show a pure prediction, but one shaped by the need to tell—and to sell—a good story.

## 5 Conclusion

I have followed the yellow brick road and am thrilled with where it has taken me. The 60-some years since I first visited Oz have taken my career to places and topics that would have baffled and surprised not only me as a child but me as an undergraduate, law student, or young lawyer. I love my work for its fascination and for the hope that, sometimes, it may help prevent problems arising from the use of bioscience. I hope that this short article may help some other authors find their own paths between science fiction and ELSI.

## Data Availability

The original contributions presented in the study are included in the article, further inquiries can be directed to the corresponding author.

## Footnotes

1 Although I have published one article that verged on literary criticism about a science fiction classic—Frankenstein (Greely, 2020).

2 Wikipedia is a useful source for an overview of “Science Fiction Studies”: its history, main authors, journals, and conferences (Wikipedia Science Fiction Studies).

3 As I have written about both chimeras and gene editing, I have referenced this story often, although I do have to note that there are parts of it that, today, induce cringes for what can be read as racist overtones, whether intended as such in 1947 or not.
